# Evaluation of Material Integrity Using Higher-Order Harmonic Generation in Propagating Shear Horizontal Ultrasonic Waves

**DOI:** 10.3390/ma17163960

**Published:** 2024-08-09

**Authors:** Rafał Radecki, Wiesław J. Staszewski

**Affiliations:** AGH University of Krakow, Faculty of Mechanical Engineering and Robotics, Department of Robotics and Mechatronics, al. Mickiewicza 30, 30-059 Krakow, Poland; rafal.radecki@agh.edu.pl

**Keywords:** ultrasonic shear waves, nonlinear wavefield, higher harmonic generation, material degradation, fatigue cracks, piezoceramic transducers

## Abstract

Material nonlinearity is explored for the assessment of structural integrity. Crack–wave interactions are of particular interest. The major focus is on higher-order harmonics, generated in propagating shear horizontal (SH) waves. These harmonics are generated due to global material nonlinearity and local effects such as fatigue cracks. The theoretical background of the proposed method is explained. The method is examined using numerical simulations and experimental tests. The former involves the Local Interaction Simulation Approach (LISA), implemented for the nonlinear shear horizontal wavefield. The latter is based on a high-frequency shear excitation approach. Experimental tests are conducted using a series of beam specimens with fatigue cracks. Low-profile, surface-bonded piezoceramic shear actuators are used for excitation. The excitation frequency is selected to minimize the number of generated modes in the examined specimens. Nonlinear ultrasonic responses are collected using a non-contact laser vibrometer. The results show that higher-order harmonic generation—based on shear horizontal wave propagation—can be used for crack detection in the presence of global material nonlinearity.

## 1. Introduction

Structures used in engineering applications require the regular monitoring of their state, whether it be material defects, degradation or structural damage, to maintain safe operation and minimize overall costs. This requirement has led to the need to develop reliable methods of material evaluation and early detection of structural defects. Methods based on the propagation characteristics of ultrasonic waves have a well-established place among many available approaches. The analyses of the attenuation, reflections and scattering have led to the development and application of the methods used in field testing [1]. It has allowed for the use of guided ultrasonic waves and their propagation characteristics for material testing and damage detection in many structures, i.e., plates [2], thick-hollowed cylinders [3] or composites [4] to name a few.

In recent years, however, nonlinear characteristics of ultrasonic waves have drawn more research interest. Although the methods based on those features are more difficult for practical applications, they offer much higher sensitivity to early-stage defects, especially at a microstructural level [5,6,7]. Overall, nonlinear features can be imposed over the propagating ultrasonic waves as a result of interacting with the following sources: material, i.e., inclusions, elastic and attenuation properties and grain structure; defects, i.e., cracks and delaminations; structural assemblies, i.e., joint friction; and intrinsic effects, i.e., overloads in the instrumentation chains. However, the last case relates to undesired experimental errors. In this paper, the first two sources, namely the material and defects, are of particular interest.

With such a range of nonlinear sources interacting with the propagating ultrasonic waves, a diversity of Non-Destructive Testing (NDT) and Structural Health Monitoring (SHM) approaches have been developed and deployed, represented by those using higher-order [8,9] or sub- [10] harmonics, mixed-frequency responses [11], shifts in resonance frequency [12], vibro-acoustic modulations [13] and the Luxembourg–Gorky effect [14]. All the abovementioned methods have been reviewed comprehensively in [15]. This work focuses on the higher-order harmonic generation in the guided ultrasonic waves. It has been evidenced as an energy transfer from the fundamental frequency $\omega_{0}$ to the integer multipliers of fundamental frequency ($2\omega_{0},\;3\omega_{0}$ and so on). However, depending on the type of the chosen guided ultrasonic wave, different results can be obtained. If one uses Lamb waves to examine chosen structures, all higher harmonics should be present in the frequency response upon the interaction with the nonlinear source [16]. However, if one focuses on the structure examination using the shear horizontal (SH) waves, due to the propagating characteristics of the aforementioned waves, only odd harmonics will be inflicted into the frequency response collected from the structure [17]. The propagation characteristics of the latter type of guided ultrasonic waves are examined in the present work.

In the last decade, more intensified research development can be observed focusing on the nonlinear features inflicted over the propagating SH waves. However, very few works have considered the third-harmonic generation of the fundamental SH_0_ wave mode. Li et al. [18] investigated the cumulative characteristic of the third-harmonic generated due to the interaction of the fundamental SH_0_ wave mode with the nonlinear material. Similar work was conducted by Wen et al. [19], but in this case, material nonlinearity was enhanced by imposing thermal degradation over the investigated material. None of the abovementioned works considers a combination of the sources, i.e., nonlinear material and frictional motion of the crack. Osika et al. [20] presented the numerical investigation of the combination of the two aforementioned sources interacting with the propagating SH_0_ mode; however, no experimental results were shown. The present work aims to fill those gaps. Other works have either focused on the generation of the second-harmonic SH_0_ mode due to the interaction of Lamb waves with nonlinear sources [21] or wave mixing [22].

The aim and novelty of the present work are the results of the interaction of the fundamental guided shear horizontal wave mode—SH_0_—with two nonlinear sources, i.e., nonlinear material and fatigue cracks executing frictional motion. A numerical study is prepared based on the Local Interaction Simulation Approach (LISA)—a finite-difference approach developed for considering sharp interface changes. The sources are considered separately and then combined for the final case. This numerical study is followed by an experimental study. In both investigational approaches, a location of the fatigue-generated crack is possible to point out in the presence of the material nonlinearity. The nonlinear gamma—γ—parameter is introduced to enhance the location of the damage in the examined structure.

The paper is organized as follows. Shear horizontal wave theory is presented in the next section. The linear and nonlinear material definitions are considered. This is followed by the description of the local type of nonlinearity, namely Coulomb’s frictional law. Section 3 introduces the numerical tool used in this work—LISA—followed by the definition of the model and the results of the investigated numerical cases. Section 4 concerns the experimental study of the problem. Setup is presented followed by the results obtained from this study. Finally, conclusions are provided in Section 5.

## 2. Shear Horizontal Waves

### 2.1. Linear Theory

**Figure 1:**
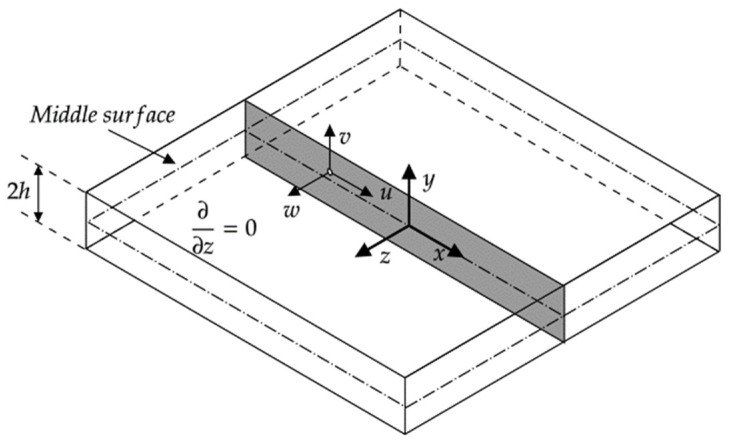
Geometric representation of the considered system and used coordinate description.

A 2h thick semi-infinite plate is considered, as illustrated in Figure 1. Here, displacement vectors corresponding to individual particles are described by the Cartesian coordinate system. The invariance along the z axis (i.e., ∂/∂z=0) is assumed for simplicity. With this assumption, wave propagation in the analyzed plate can be described using Navier’s elastodynamic equation

(1) $$\rho =\nabla \sigma +\tilde{F},$$

where ρ is the material density, $W={\left[u,v,w\right]}^{T}$ is the vector field of particle displacement, t is time, σ is the stress tensor and $\tilde{F}$ is the body force vector. Considering the linear, homogenous and isotropic material, the above set of equations can be rearranged to the linear Navier’s displacement equations [2,23]

(2) $$\mu \nabla^{2}W+\left(\lambda +\mu \right)\nabla \nabla W=\rho \frac{\partial^{2}W}{\partial t^{2}},$$

where λ, μ are the Lamé constants. Following the representations shown in Figure 1, Equation (2) describes the motion of the Longitudinal and shear waves. Assuming that the propagation direction goes along the x axis, the particle motion of the former type goes along that axis. For the latter, further differentiation is needed. Shear wave particle motion is oriented perpendicular to the propagation direction. These waves can be polarized horizontally—along the z axis—and vertically—along the y axis. As a result, the former are known as shear horizontal (SH) waves, whereas the latter as Shear Vertical (SV) waves. The fundamental mode of SH—i.e., the SH_0_ wave mode—is particularly attractive for structural damage detection applications due to its non-dispersive nature and good sensitivity to microstructural damage.

Assuming invariance along the z axis—∂/∂z=0, the particle motion for the SH waves is obtained by simplifying Equation (2) to the following form

(3) $$\frac{\partial^{2}w}{\partial x^{2}\;}+\frac{\partial^{2}w}{\partial y^{2}\;}=\frac{1}{c_{T}^{2}}\frac{\partial^{2}w}{\partial t^{2}\;},$$

where w is the only non-zero displacement component and $c_{T}=\sqrt{\mu /\rho }$ is the bulk shear wave speed. Next, the non-zero displacement component w is assumed as a harmonic function in the form of

(4) $$w\left(x,y,t\right)=f\left(y\right)e^{i\left(kx\;-\;\omega t\right)},$$

where ω is the circular frequency, k=2π/l is the wavenumber and l is the wavelength; in this case, the notation was changed when compared to the literature [23] to avoid confusion with the Lamé constant introduced earlier. Substituting Equation (4) to Equation (3) yields

(5) $$\frac{\partial^{2}f\left(y\right)}{\partial y^{2}}+\left(\frac{\omega^{2}}{c_{T}^{2}}\;-\;k^{2}\right)f\left(y\right)=0.$$

The general solution of this equation can be given as

(6) $$f\left(y\right)=Asin\left(qy\right)+Bcos\left(qy\right),$$

where

(7) $$q=\sqrt{\frac{\omega^{2}}{c_{T}^{2}}\;\;-\;k^{2}},$$

and *A* and *B* are arbitrary amplitudes.

Assuming that for the plate of d=2h thickness, the upper and lower surfaces (y=±h) are traction-free, the boundary conditions reflecting that state are given as

(8) $${\left.\sigma_{yz}\left(x,\;y,t\right)\right|}_{y=\pm h}=0.$$

By imposing this boundary condition on Equation (4), the characteristic equations can be obtained as

(9) $$\sin\left(qh\right)=0\;\mathrm{and}\;\cos\left(qh\right)=0,$$

for the symmetric and antisymmetric wave components, respectively. Knowing that $\sin\left(m\right)=0$ when m=nπ(n∈{0,1,2,…}) and $\cos\left(m\right)=0$ when $m=n\pi /2(n\in \left\{1,3,5,\ldots \right\})$, the solutions to Equation (9) are obtained in the form

(10) qh=nπ/2,

where $n\in \left\{0,2,4,\ldots \right\}$ for symmetric and $n\in \left\{1,3,5,\ldots \right\}$ for antisymmetric modes. Thus, the displacement description given by Equation (4) can be decomposed into symmetric and antisymmetric components—with respect to y—as

(11) $$w^{s}\left(x,y,t\right)=B\cos\left(qy\right)e^{i\left(kx\;-\;\omega t\right)}\;\;\mathrm{and}\;\;w^{a}\left(x,y,t\right)=A\sin\left(qy\right)e^{i\left(kx\;-\;\omega t\right)},$$

respectively.

Next, through the substitution of Equation (7) to Equation (10), the following formula is obtained as

(12) $$\frac{\omega^{2}}{c_{T}^{2}}-\frac{\omega^{2}}{c_{P}^{2}}={\left(\frac{n\pi }{2h}\right)}^{2}.$$

For ω=2πf, the above equation can be solved for the phase velocity $c_{P}$—as a function of the frequency–thickness (fd) product—giving

(13) $$c_{P}\left(fd\right)=\pm 2c_{T}\left\{\frac{fd}{\sqrt{4{\left(fd\right)}^{2}-n^{2}c_{T}^{2}}}\right\}.$$

For *n =* 0, the solution of the above equation corresponds to the SH_0_ mode. One can note that in this case $c_{P}=c_{T}$; thus, a non-dispersive wave propagates with the bulk shear wave speed $c_{T}$. Analogically, the relevant group velocity can be obtained as

(14) $$c_{g}\left(fd\right)=c_{T}\sqrt{1-\frac{{\left(n/2\right)}^{2}}{{\left(fd/c_{T}\right)}^{2}}}\;\;\;\;\;\;\;\;\;\;\;\;\;\;\left(fd\geq {\left(fd\right)}_{n}\right).$$

By analyzing the above equation, it can be clearly noticed that at the cut-off frequencies—$f_{co}=\left(c_{T}q\right)/\left(2\pi \right)$—the group velocity of any mode is equal to zero, and as fd approaches infinity for any given n, the group velocity of any SH mode approaches the velocity of bulk shear wave $c_{T}$. Moreover, Equations (13) and (14) also show that the SH_0_ mode, which is of particular interest in the present work, is non-dispersive.

### 2.2. Nonlinear Theory—Global Nonlinearity

As indicated in the introduction, material nonlinearity can be one of the sources of inducing higher-order harmonics in the propagating guided shear horizontal wave. As it was shown in [5,24], a part of the energy of propagating SH waves will transfer to the third and higher-order odd harmonics due to continuous interactions between the aforementioned wave and the nonlinear material. Research on this physical effect for SH waves has undergone some extensive development, showing the existence of nonlinear wave modes [17]. Multiple mathematical models were proposed in the form of constitutive equations in order to characterize general material nonlinearity [25]. Landau and Lifshitz introduced one of these models in [26]. In the scope of the present work, fourth-order expansion was adopted to characterize the nonlinear medium. An energy density U(E) equation extended to the fourth order takes the form of

(15) $$U\left(E\right)=\frac{\lambda }{2}I_{1}^{2}+\mu I_{2}+\frac{A}{3}I_{3}+BI_{1}I_{2}+\frac{C}{3}I_{1}^{3}+EI_{1}I_{3}+FI_{1}^{2}I_{2}+GI_{2}^{2}+HI_{1}^{4},$$

where λ and μ are the Lamé constants; A, B and C are the third-order elastic constants (TOECs) and E, F, G and H are the fourth-order elastic constants (FOECs) [27]. The scalars $I_{1}$, $I_{2}$ and $I_{3}$ denote first, second and third invariants of the Green–Lagrange strain tensor E, which is defined as

(16) $$E=H+H^{T}+H^{T}H,$$

where H is the gradient of displacement field of particles. To obtain the wave equation in a nonlinear form, the components of a second Piola–Kirchhoff stress tensor should be calculated as

(17) $$S_{ij}=\frac{\partial U\left(E\right)}{\partial {\left(E\right)}_{ij}},$$

where $i,j\in \left\{x,y,z\right\}$. The substitution of Equations (16) and (17) into Equation (1) leads to obtaining a nonlinear wave motion equation, which was then implemented in a numerical model based on the Local Interaction Simulation Approach. This implementation will be discussed in the following section.

### 2.3. Nonlinear Theory—Local Nonlinearity

**Figure 2:**
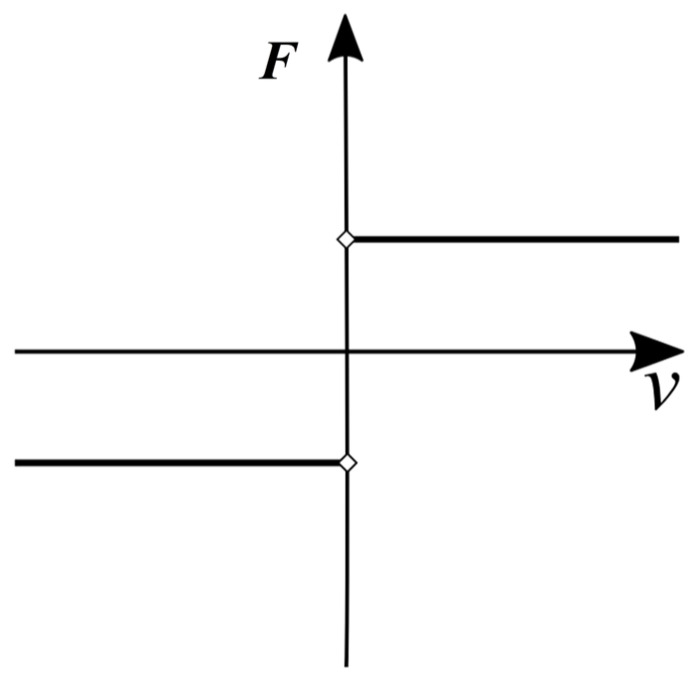
Graphical representation of Coulomb’s frictional law.

The second source of the nonlinear phenomenon considered in this work is the shear stick–slip movement of fatigue crack surfaces. Only the shear movement of crack surfaces perpendicular to the SH wave propagation direction is considered in this work. As the propagating SH wave approaches the location of the damage, the faces of the crack interact mechanically via the friction force F, which results from the contact between asperities under a normal force (pressure). The Coulomb friction formulation with a stick–slip behavior [28] is implemented with an arbitrary assumed distribution of the pressure acting on crack surfaces. The value and sign of the friction force are dependent on the value of the relative velocity between the crack surfaces as indicated in Figure 2.

## 3. Numerical Simulation

### 3.1. Local Interaction Simulation Approach—LISA

The LISA method was first introduced in the 90s of the previous century by Delsanto et al. [29,30,31] for the purpose of modeling wave propagation in media with sharp interfaces and inclusions of different material properties. This approach has also been used extensively for simulating crack–wave interactions of the propagating Lamb wave used for damage detection investigations [32,33]. The work in [16,28] presents the application of LISA for the modeling of Lamb wave propagation in a hyperelastic medium. This approach is based on the finite-difference (FD) formulation used for the discretization of the space derivatives in the governing differential equations. The explicit central difference formula is used for time-domain discretization. Thus, the method is well suited for parallel computations, as demonstrated in [34]. For a medium described by two Lagrangian coordinates, the LISA method discretizes the geometrical models into a 2-D grid of rectangular cells. Material properties are assumed to be constant within cells but they may differ among these cells. As a result, LISA is an appropriate tool for wave propagation in complex media that are heterogeneous, anisotropic and nonlinear [30]. The implementation of the LISA framework for SH wave propagation in a hyperelastic medium follows that given in [28]; therefore, only a short description is presented below.

**Figure 3:**
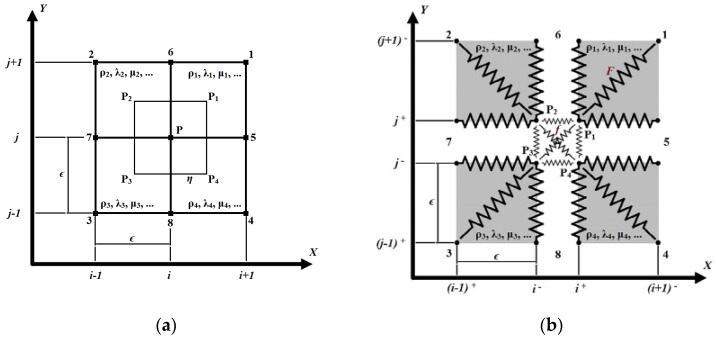
Discretization approaches (**a**) for nonlinear material definition; (**b**) for frictional interface motion.

To obtain the iteration equations for nonlinear LISA, the spatial derivatives in Equation (1) are replaced by difference formulas. For a two-dimensional case, the iteration equations are derived for the point at the intersection of four adjacent cells—point *p* shown in Figure 3a. In this derivation, each cell is treated separately, and nodal point *p* is replaced by four points $P^{\left(i\right)}$ as shown in Figure 3b, where $i\in \left\{1,2,3,4\right\}$. The Navier elastodynamic equation—evaluated at the introduced points $P^{\left(i\right)}$—can be written as follows.

(18) $$\frac{2\Delta \sigma_{xz}^{\left(i\right)}}{\Delta x}+\frac{2\Delta \sigma_{yz}^{\left(i\right)}}{\Delta y}≃\rho^{\left(i\right)}\frac{\partial^{2}w}{\partial t^{2}}\;$$

The finite-difference formulas $\Delta \sigma_{xz}^{\left(i\right)}$ and $\Delta \sigma_{yz}^{\left(i\right)}$ are evaluated for assumed stress component distributions for each cell 1–4 presented in Figure 3b. To complete the set of four equations defined by Equation (18), a stress continuity needs to be enforced between the neighboring cells. This leads to the reduction in stress tensor components in Equation (18). After summing the equations from all four cells with the imposed stress continuity, the stress-based iteration equation for nodal point *P* can be obtained as

(19) $$\frac{2\left(\sigma_{xz}^{\left(1\right)}-\sigma_{xz}^{\left(2\right)}-\sigma_{xz}^{\left(3\right)}+\sigma_{xz}^{\left(4\right)}\;\right)}{\Delta x}+\frac{2\left(\sigma_{yz}^{\left(1\right)}+\sigma_{yz}^{\left(2\right)}-\sigma_{yz}^{\left(2\right)}-\sigma_{yz}^{\left(4\right)}\right)}{\Delta y}\;≃\sum_{i}\left(\rho^{\left(i\right)}\right)\frac{\partial^{2}w}{\partial t^{2}}$$

It should be noted that the presented derivation using the LISA scheme is based on evaluating the elastodynamic equation and imposing continuity of selected stress tensor components across the interfaces of adjacent cells. As a result, this procedure can be conducted for the assumed form of the constitutive elastic relation and the geometrical definition of strains.

Due to the action of decomposing the point *p* into four separate points, one for each cell, it is possible to model the stick–slip motion of the crack interfaces using the LISA framework. These nodes are grouped in pairs and are considered independent. First, the classic iteration equations are composed for each node as it was shown previously. Then, for node pairs, which are on the same surface of the crack, the constraints are imposed for the stick motion in the following form

(20) $$w_{t+1}^{k-}-w_{t}^{k-}=w_{t+1}^{k+}-w_{t}^{k+}.$$

where w is the displacement along the z axis, with superscripts corresponding to the kth node at the location of the defined crack and subscripts $\left\{t,t+1\right\}$ corresponding to the current and next time step of the simulation. The signs $\left\{-,+\right\}$ represent the surfaces of the crack, for instance, left and right.

Using the Lagrange multipliers method, it is possible to implement the abovementioned constraints, as well as determine crack node displacement $w_{t+1}^{k-}$, $w_{t+1}^{k+}$ and reaction body forces ${\tilde{F}}_{r}^{\left(k-\right)}=-{\tilde{F}}_{r}^{\left(k+\right)}$. During each time step of the simulation, within each node pair, the values of the module of reaction body forces $\left|{\tilde{F}}_{r}^{\left(k-\right)}\right|$ are compared with the body force equivalent ${\left({\tilde{F}}_{sf}^{\left(k\right)}\right)}_{max}$ of the maximum static friction force. If $\left|{\tilde{F}}_{r}^{\left(k-\right)}\right|>{\left({\tilde{F}}_{sf}^{\left(k\right)}\right)}_{max}$, then nodes at the location of the crack will move independently, i.e., in slip motion. Moreover, an additional external body force equivalent ${\tilde{F}}_{kf}$ of the kinematic friction force is imposed on each of these nodes. Its direction and sense are dependent on the direction and sense of the relative velocity vector of the considered node pair.

### 3.2. Model Description

**Figure 4:**
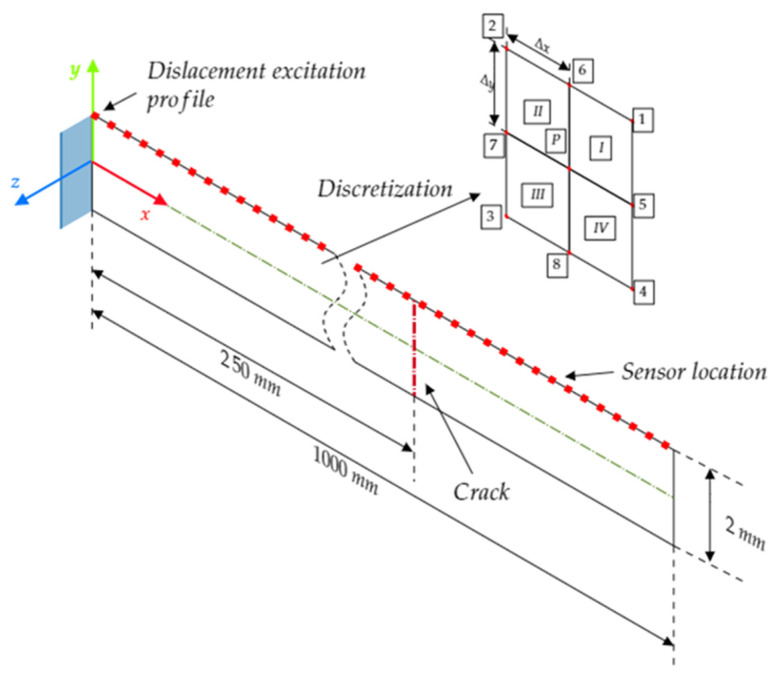
Graphical representation of the numerical model.

To scrutinize the influence of a nonlinear medium and its interaction with local defects on the propagation characteristics of the SH wave, a numerical model of the aluminum beam was prepared. A 2 mm thick beam was considered with its length equal to 1000 mm as shown in Figure 4. Such a length was chosen to avoid any reflection of the wave from the opposite end to the excitation point. The model was discretized into a set of square elements, with a size of Δx=Δy=0.1 mm. This setup led to obtaining 20 elements through the thickness of the considered beam. The time step for the simulations was set as 10 ns to maintain the numerical stability during calculations. The material properties of the model are presented in Table 1. The Landau TOECs and FOECs values were calculated using the data presented in [35].

The local type of nonlinearity in the form of the crack was located 250 mm from the left side of the beam. As the frictional motion of the crack interfaces is investigated, the static and kinetic friction coefficients were set to $\mu_{s}=0.5$ and $\mu_{k}=0.4$, respectively. Six depths of the fatigue crack were investigated as the percentage of the beam thickness (from 0 to 25% with a step every 5%). For the numerical cases, where fatigue crack was considered, a distribution of compressive residual stress $\sigma_{xx}(y)$ was assumed over the crack interfaces. These stresses may result from manufacturing processes such as cold rolling. Following the work presented in [36], a symmetrical stress distribution with respect to the center of the beam thickness was imposed. For $y\in \;\langle 0,0.25\rangle \;mm$, the residual stresses were described by $\sigma_{xx}\left(y\right)=\alpha y+\beta$, where $\alpha =32\frac{MPa}{mm}$ and β=−10 MPa. By imposing the residual stresses over the crack surfaces, it was possible to determine the body forces between equivalents of static and kinetic frictional forces.

A fifteen-cycled sine signal multiplied by the Hanning window was used as an excitation signal. The central frequency was set as 200 kHz. The excitation signal was assigned to the left side of the beam as a displacement boundary condition and distributed uniformly over the beam thickness. The amplitude was equal to 1 μm. The choice of the excitation frequency and the thickness of the modeled beam led to the excitation of only the fundamental SH wave mode, i.e., SH_0_ mode. Its non-dispersive characteristics significantly simplified the data analysis and interpretation of numerical results. Finally, the responses of the modeled structures were collected as displacements from the upper surface of the beam. Measurement points were selected over the entire length of the model with a step of every 20 mm.

### 3.3. Results

**Figure 5:**
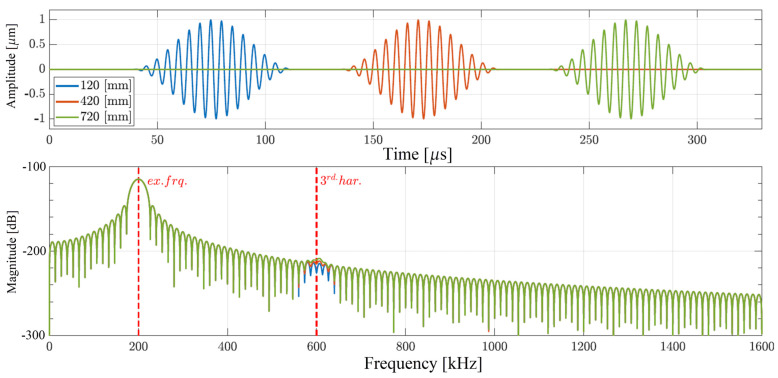
Structural responses obtained from the numerical simulations when only nonlinear material is considered: (**top**) time-domain signals; (**bottom**) frequency-domain signals.

First, only the nonlinear material is considered. Results of the simulation presented in Figure 5 are captured at three measurement points: 120 mm, 420 mm and 720 mm from the excitation point. In the time domain, only one non-dispersive wave packet is visible (for each point) that corresponds to the primary non-dispersive SH_0_ mode. In the frequency domain, apart from the fundamental excitation frequency, one can observe the generation of the third harmonic. Moreover, the amplitude of this higher-order component increases with distance as shown in the bottom plot of Figure 5. Due to the non-dispersive characteristic of the SH_0_ mode, the internal response between the first and third harmonics is not dependent on the chosen frequency. Thus, at any chosen frequency, when only the SH_0_ wave mode is analyzed, the cumulative effect with the propagation distance is observed due to the presence of global-type nonlinearity in the form of the nonlinear material definition based on the Landau material description.

**Figure 6:**
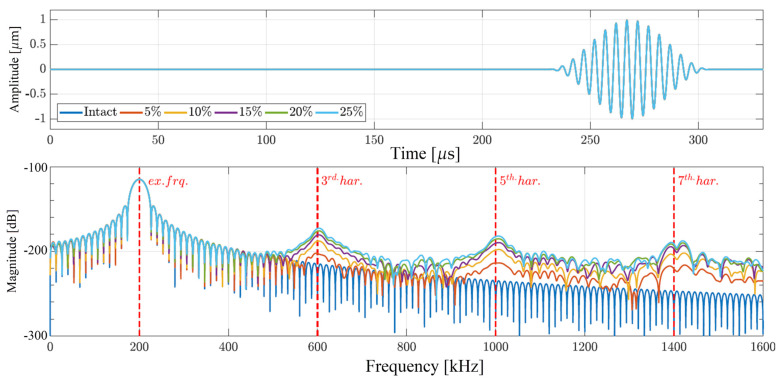
Structural responses obtained from the numerical simulations when only the frictional motion of the crack surfaces is considered: (**top**) time-domain signals; (**bottom**) frequency-domain signals.

The second case is focused on the interaction of the propagating wave with a local type of nonlinear source, namely the shear movement of crack surfaces. This motion was implemented through the incorporation of Coulomb’s friction model in the scope of the LISA model. The results of the performed simulations are presented in Figure 6. The time-domain signals are collected from the measurement point at 720 mm from the excitation site for six different depths of damage. As can be seen in the top plot of Figure 6, no significant difference can be observed among the wave packages obtained from the examined beam with different levels of damage. However, after transferring to the frequency domain, significant differences can be observed due to the increasing depth of the crack. As shown in the bottom plot of Figure 6, the magnitude of the generated higher-order harmonics is proportional to the depth of the crack. Finally, when comparing the bottom plots of Figure 5 and Figure 6, it can be observed that the local type of nonlinearity, i.e., the frictional motion of the crack surfaces, generates much higher magnitudes and numbers of the higher-order harmonics upon its interaction with the propagating wave than the global type of nonlinearity being the nonlinear material definition.

**Figure 7:**
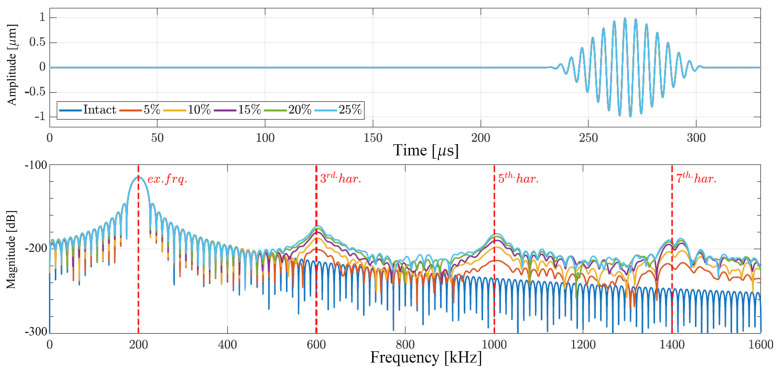
Structural responses obtained from the numerical simulations when both nonlinear material and frictional motion of the crack surfaces are considered: (**top**) time-domain signals; (**bottom**) frequency-domain signals.

In the last case, both nonlinear sources are implemented, and the results of such simulations are presented in Figure 7. Similar to the case of only local nonlinearity, the displacement responses in the time and frequency domains from the measurement point are located at a distance of 720 mm from the excitation. No visible changes can be observed in the time-domain signals due to the increase in the depth of the frictional crack. However, the opposite can be said about the frequency representation of the analyzed signals.

As in the case where only the local type of nonlinearity is defined in the examined model, for both types of nonlinearities present, higher-order harmonics can be noticed with significant levels of magnitudes. Furthermore, following a deeper analysis, one can also observe that the levels of magnitude in the case of both types of nonlinearities present are higher than when only frictional crack motion is considered. This observation could lead to the conclusion that a certain level of the superposition of the higher harmonics generated from the nonlinear material and frictional motion of the crack has occurred.

**Figure 8:**
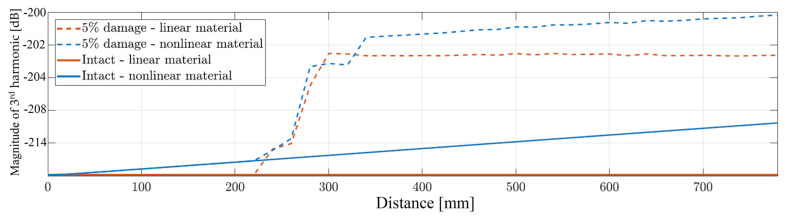
Third-harmonic magnitude over the propagation distance from the models with different combinations of nonlinear sources.

To confirm the abovementioned statement, all collected signal responses have been analyzed from the perspective of the generated third higher harmonic for the following cases: an intact beam with the linear material definition; an intact beam with the nonlinear material definition; a damaged beam (frictional motion of the crack surfaces) with linear material definition and a damaged beam (frictional motion of the crack surfaces) with nonlinear material definition. Obtained magnitudes of the third harmonics are plotted in the propagation distance domain and presented in Figure 8. It can be observed that in the case of the intact beam with linear material definition, no third harmonic is visible. The value obtained from the frequency representation remains constant through the whole distance of wave propagation. Next, when the beam with the nonlinear material definition is scrutinized, a linear relationship between the third-harmonic magnitude and propagation distance can be seen. This observed phenomenon corresponds to the synchronism between the first and third harmonics, causing a continuous power transfer between the two harmonics—due to the presence of the global type of nonlinearity—and agrees with the available literature as well [18,19]. The third curve from the bottom refers to the model with the linear material definition and the presence of the crack with induced frictional motion among the asperities. One can observe that as the wave approaches the vicinity of the crack, a high-level of magnitude third harmonic is generated upon the crack–wave interaction. As the wave propagates further from the position of the damage, the value of the magnitude remains almost constant with respect to the propagation distance. When both types of nonlinearity are present in the analyzed numerical model, an increase in the third-harmonic magnitude can be seen in the area before the crack. Then, upon the interaction of the wave with the friction interfaces, a significant increase is observed, which is followed by the linear increase in the magnitude with the propagation distance in the area after the damage.

The observations obtained from the analysis of the results presented in Figure 8 confirm that in the case of the SH_0_ wave mode propagation, a superposition of the third harmonics generated from different nonlinear sources is possible to obtain. In the next section, an experimental validation is presented to confirm the numerical findings.

## 4. Experimental Investigation

### 4.1. Setup

**Figure 9:**
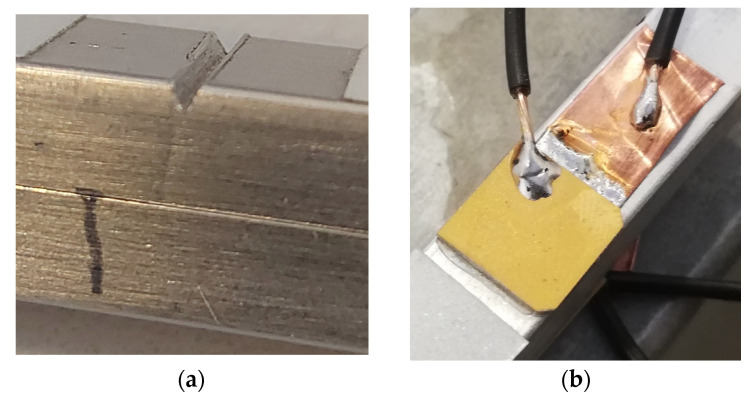
Aluminum beam setup used for the experimental investigations: (**a**) 5 mm deep crack measured from the notch; (**b**) low-profile Noliac CSAP03 shear plate actuator.

**Figure 10:**
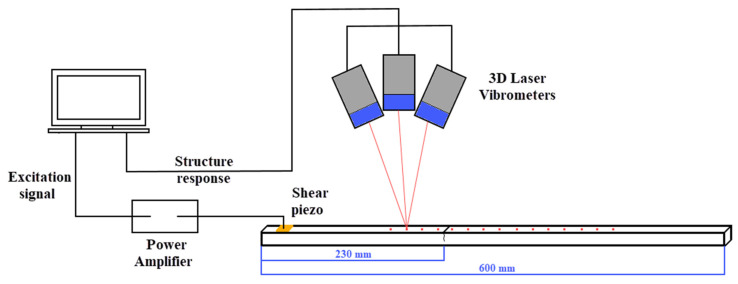
Schematic diagram showing the overall experimental arrangement and 3-D orientation of measurement.

**Figure 11:**
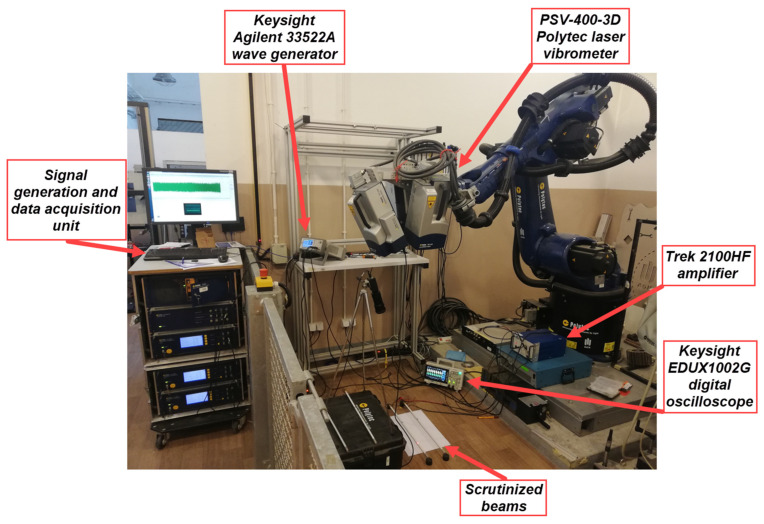
Picture of the experimental setup. Beams in the bottom are supported using rubber stands.

The investigated phenomena were validated experimentally using aluminum beam specimens. Two 10 × 10 × 600 mm beams made of PA38 (AW-6060) material were used—with and without damage. One of the components was fatigue-cycled to obtain the crack as shown in Figure 9a. A three-point bending technique was utilized for the fatigue loading, where 75,000 cycles led to obtaining a 5 mm deep crack positioned 230 mm from one of the ends of the beam as shown in Figure 10. A notch was imposed over the undamaged beam so that the only difference between the beams would be that of the fatigue-induced crack. A low-profile Noliac CSAP03 (Kvistgaard, Denmark) shear plate actuator was mounted on the beams using cyanoacrylate glue as shown in Figure 9b. A copper foil was used to wire the bonded bottom of the transducers. The beam specimens were freely supported at the edges when the experimental tests were performed. Small rubber tubular elements were used for the supports, as depicted in Figure 11.

The experimental arrangements used in the present work are illustrated in Figure 10 and depicted in Figure 11. A shear plate actuator was used for the tone-burst ultrasonic wave excitation. The excitation signal was generated using a Keysight Agilent 33522A arbitrary wave generator. Before transmitting to the actuator, the tone-burst signal was magnified using a Trek 2100HF amplifier. To reduce the number of generated wave modes a ten-cycled sine signal multiplied by the Hanning window with a center frequency of 70 kHz was used as the excitation signal. The amplitude of the propagating wave was set to 400 V_pp_. A two-channel Keysight EDUX1002G digital oscilloscope was used to monitor the excitation signal to guarantee an undistorted transmission. Response data were gathered using a PSV-400-3D Polytec laser vibrometer. Laser beams from three independent vibrometer heads were focused on one point, allowing for in-plane and out-of-plane velocity measurements in the X, Y and Z directions (Figure 10). Since shear horizontal waves in the beam were generated, measurements in the in-plane Y direction were of major interest. Altogether, 15 points on the beam—separated by 22 mm—were selected for measurements. The measurement at each point is the average of 10 signals, which allowed us to obtain a higher signal-to-noise ratio. The crack was positioned between the 4th and 5th measurement points, as shown in Figure 10.

### 4.2. Results

**Figure 12:**
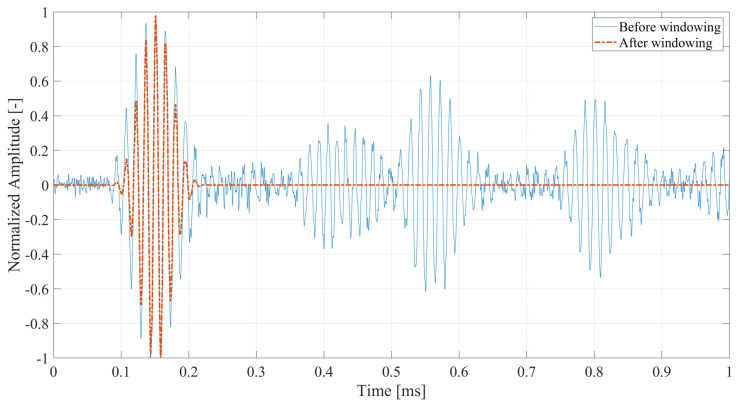
Time-domain response collected from the undamaged beam at the location of the 7th measurement point. Representation before and after windowing.

**Figure 13:**
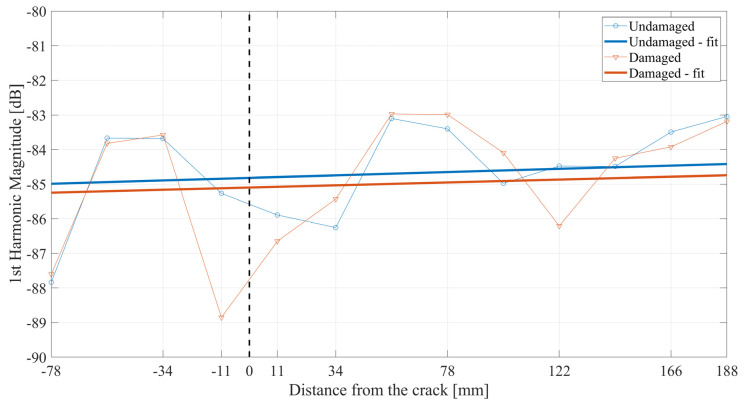
First-harmonic magnitudes over the distance from the crack.

As mentioned in the previous section, the experiments were performed on beam structures, resulting in more complex structural responses than the ones obtained in the numerical investigation. For that reason, the preprocessing of the received signals is necessary before analyzing the generated higher harmonics. An example of the signal is shown in Figure 12. This signal is collected from the seventh measurement point from the undamaged beam. The amplitude of the signal was normalized with respect to the highest value available in the signal. Due to the fact that for the chosen central frequency, the SH_0_ wave mode has the highest group velocity in the Y direction of the measurement, the first package will correspond to that wave mode. Any additional packages in the signal correspond to the wave mode conversion and/or reflection due to the interaction with the boundaries of the examined specimen. Thus, to analyze the frequency representation of only the SH_0_ wave mode, the windowing of the first package is performed on all structural responses. The Hanning window is used for this purpose. The results of the windowing are available in Figure 12 as well. This approach leads to the proper analysis of the first and higher harmonics, which strictly correspond to the SH_0_ wave mode generated upon the first interaction with the nonlinear source. First, the first-harmonic magnitudes are collected and displayed in Figure 13 in the domain of the distance from the crack. The black dashed line refers to the position of the crack and is marked on the following figures.

Very small changes in the first-harmonic magnitude can be observed with the propagation distance, which may be the effect of averaging the structural responses during the measurement. Linear curve fitting was applied to the obtained results to point out the trends in the magnitude changes in a clear manner. Although the real data vary strongly, when compared to the fitted curves, the differences between the values are very small. The variation in the collected data might be the result of averaging the measured signals.

When analyzing the first-harmonic magnitudes, one can observe a high change in the magnitude between the damaged and undamaged beam in the vicinity of the crack’s location.

**Figure 14:**
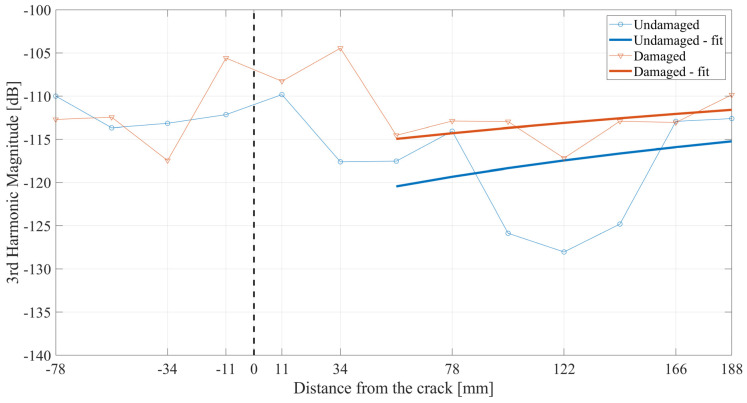
Third-harmonic magnitudes over the distance from the crack.

In Figure 14, third-harmonic magnitudes are presented from both examined structures. It can be noticed that before the interaction of the wave with the crack, the magnitudes from both beams are of similar value levels. Upon the interaction with the frictional crack, higher values of the third harmonic are obtained from the damaged beam when compared to the undamaged one. The drop in the values in the area after the crack may correspond to the damping of the wave with the propagation distance. However, through the application of the curve fitting to the part of the plots after the crack, an increase in the magnitudes can be observed. Moreover, a clear difference between the undamaged and damaged beams is obtained. For the damaged beam, the values of the real data oscillate with respect to the fitted curve and the differences are at an acceptable level. However, in the case of the undamaged beam, the differences are of a higher level, especially for the 9th to 11th measurement points. This phenomenon may be caused by the local damping of the signal.

**Figure 15:**
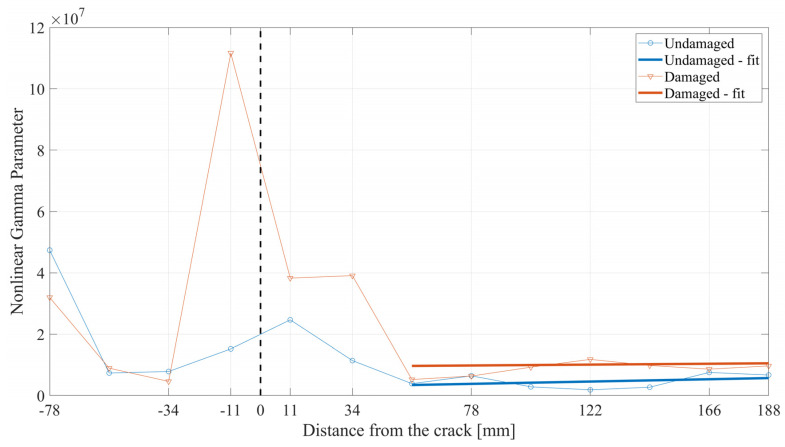
Nonlinear γ parameter over the distance from the crack.

**Figure 16:**
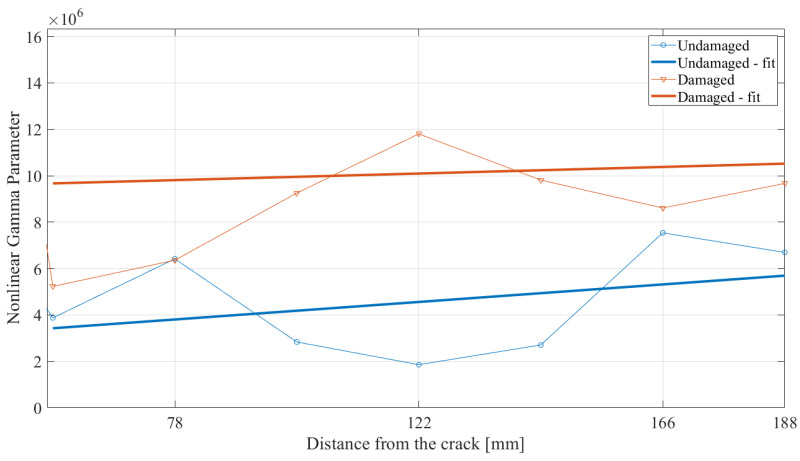
Nonlinear γ parameter over the distance from the crack. The area after the cracks’ location.

The final analysis concerns defining a nonlinear parameter that considers the changes occurring in both the first and third harmonics and is defined as

(21) $$\gamma =\frac{A_{3}}{A_{1}^{3}},$$

where $A_{3}$ and $A_{1}$ are the third- and first-harmonic magnitudes, respectively. The results of the calculated gamma parameter are shown in Figure 15. Using this approach, a clear difference is obtained between the curves when analyzing the parameter values in the area of the crack. Significantly higher values of the γ parameter have been obtained for the damaged beam than the undamaged one. Moreover, by analyzing the variation in this parameter over the whole length of the beam, it is possible to narrow the area in which a possible location of the crack is much higher. This finding can be used to localize the fatigue-generated crack using the nonlinear propagation characteristics of guided SH waves. Finally, by analyzing the area after the damage, small changes are obtained. However, it is still possible to differentiate the values of the parameters corresponding to the damaged and undamaged beams. For a deeper analysis, the area from 56 mm to 188 mm is enlarged and presented in Figure 16.

By analyzing the γ parameter in the area after the crack, an increase in the value with the propagation distance can be observed for both examined specimens, which corresponds to the cumulative effect observed in the numerical study and in the literature [18,19]. Moreover, when comparing the value of the fitted curves, the γ parameter is more than two times higher for the damaged beam from the values collected than the undamaged beam. This difference can also help identify the state of the beam. Considering the scale of the obtained values of parameter γ for both states of damage, the oscillations of the real data with respect to the fitted curves are the result of the obtained first- and third-harmonic magnitudes. Due to the incorporation of both harmonics, the discrepancies are much lower than in the case of the third harmonic.

## 5. Conclusions

The characteristics of the generated higher harmonics resulting from the interaction of the shear horizontal wave with different sources were investigated. This work involved numerical simulations and experimental validations. The focus was on the identification of damage using the nonlinear characteristics of the shear horizontal waves. This objective was successfully achieved with the help of numerical and experimental investigations. The major conclusions of this work can be summarized as follows.

The results from the numerical simulations confirm that the fundamental shear horizontal wave mode—SH_0_—is synchronous with the higher-order harmonics of the same mode. That leads to the presence of cumulative effects when the wave propagates in the nonlinear medium. The increase in the third harmonic with the propagation distance is confirmed. Furthermore, it is also shown that the local type of nonlinear source, i.e., the frictional motion of the cracks’ surfaces, inflicts nonlinear features upon the propagating wave. However, the magnitude of the higher harmonics does not increase with the propagation distance. When considering the presence of both nonlinear sources in the examined medium, a superposition-like behavior of the third harmonics generated from the global and local nonlinear sources is observed. Nonetheless, numerical simulations also confirm that frictional motion induced by crack–wave interactions generates higher values of the third and other higher-order harmonics than the continuous interaction of the propagating wave with the nonlinear material. Therefore, the generation of higher-order harmonics can be used to localize the fatigue-generated damage in the investigated structure in the presence of the globally distributed material nonlinearity.

This experimental investigation shows that the cumulative effect is difficult to obtain. The increase in the third-harmonic magnitude with the propagation distance is only possible to observe when a first-order curve fitting is applied. However, analyzing the third-harmonic characteristics over the propagation distance allowed us to identify the location of the fatigue-induced crack, which has a bigger influence on the generation of higher harmonics than the material’s nonlinearity, thus validating the results obtained from numerical simulations. Moreover, through the calculation of the γ parameter as the ratio of the third-harmonic magnitude to the cube of the first-harmonic magnitude, it is possible to point out the location of the damage with higher precision than in the case of analyzing only third-harmonic magnitudes. Finally, by analyzing the area after the crack, it is still possible to identify the damaged beam from the undamaged one.

In summary, the nonlinear propagation characteristics of the shear horizontal wave help identify the source of nonlinearity—global or local—and in the case of the local type, it is also possible to find its location.

Future work in the area should focus on the experimental identification of the depth of the crack. In addition, further narrowing down the location of the damage would be advantageous.

## Figures and Tables

**Table 1:** Material properties used in the numerical simulations.

Linear Material Properties	Third-Order Elastic Constants (TOECs)	Fourth-Order Elastic Constants (TOECs)
Young’s modulus E=68.9 GPa	A=−195.9 GPa	E=81.7 GPa
Poisson’s ratio ν=0.33	B=−118.3 GPa	F=165.2 GPa
$\mathrm{Density}\;\rho =2700\;kg/m^{3}$	C=−3.5 GPa	G=228.4 GPa
		H=−25.1 GPa

## Data Availability

The raw data supporting the conclusions of this article will be made available by the authors on request.
